# Falls and loneliness among Chinese older adults: evidence from a nationally representative longitudinal study

**DOI:** 10.3389/fpubh.2025.1630345

**Published:** 2025-07-15

**Authors:** Zhiqiang Yu, Meng Jiang, Yueyun Zhang

**Affiliations:** ^1^Department of Social Work, School of Philosophy and Social Development, Shandong University, Jinan, China; ^2^School of Social Sciences, Harbin Institute of Technology, Harbin, China

**Keywords:** falls, loneliness, China, older adults, social adaptation

## Abstract

**Background:**

Falls and loneliness are both common during older adulthood and detrimental to older adults’ health. However, it remains unclear whether and how falls may be longitudinally related to loneliness among older adults, despite some cross-sectional evidence in both Western and Chinese contexts. This study aimed to examine the longitudinal association between falls and loneliness among Chinese older adults, its potential variation across age groups, and the role of social adaptation in mediating the association.

**Methods:**

Data were from four waves of the China Longitudinal Aging Social Survey (CLASS, 2014–2020), and random-effects regression models were applied to a sample of 31,406 person-wave observations from 11,063 respondents. Both falls and loneliness were self-reported. Mediation analysis was conducted using the Baron and Kenny method and Bootstrapping procedures.

**Results:**

First, falls were associated with higher levels of loneliness (*p* < 0.001). Second, the association differed by age group, with its magnitude being higher for the young-old (aged 60–69) (*p* < 0.05) and the oldest-old (aged 80 and over) (*p* < 0.01) than for the old-old (aged 70–79). Third, social adaptation played a mediating role (accounting for 10% of the total effect), that is, falls resulted in decreased social adaptation which further increased loneliness.

**Conclusion:**

These findings enrich our understanding of the pivotal role of falls in shaping the psychological well-being of older adults. In terms of policy relevance, falls prevention programs should be integrated into mental health initiatives for older adults, recognizing falls not merely as physical health events but as potential triggers for loneliness.

## 1 Introduction

Loneliness is defined as a subjective and negative experience that arises from a discrepancy between an individual’s desired and actual social connections (1). In recent years, loneliness has emerged as a global public health concern (2). Older adults, in particular, have been identified as a vulnerable group prone to experiencing loneliness (3), due to the considerable reduction in social connections during later life and the accelerated aging of the population structure (4, 5). As the country with the largest older adults population size in the world, China has observed both a high and growing prevalence of loneliness among older people over the past few decades (5, 6). In terms of its consequences, loneliness has been linked to a wide range of adverse health outcomes, including depression (7), cognitive impairment (8), and mortality (9).

Falls are defined as “an unexpected event in which the participants come to rest on the ground, floor, or lower level” (10). Falls are common and represent a significant cause of morbidity and mortality among older adults. According to estimates from the World Health Organization, the annual occurrence rate of falls among socially active older adults can be around 30% (11). China is no exception in this regard. Studies based on Chinese data have found that 18.0–19.0% older adults experienced falls (12, 13). In terms of their health implications, some falls can incur significant physical injuries and disabilities (14, 15). Some falls can be even fatal. Indeed, falls have been ranked as the second leading cause of unintentional injury deaths, and older adults account for the greatest number of fatal falls worldwide (11). In consideration of their high prevalence and severe health threats, falls have increasingly become a public health concern in both developed and underdeveloped countries such as China (11).

In extending prior research on the psychological consequences of older adults falls, this study aimed to examine the longitudinal association between falls and loneliness among Chinese older adults, its age disparities, and the potential mediating role of social adaptation. While research has extensively documented the physical consequences of falls, their psychological impacts, particularly on feelings of loneliness, remain understudied despite their potential significance for older adults’ quality of life. The stereotype threat model (16) offers a valuable theoretical framework for understanding the relationship between fall experiences and loneliness among older adults. Stereotype threat occurs when individuals perceive they may be judged or treated negatively based on age-related stereotypes, leading to anxiety that can affect their performance and well-being (17). Older adults who have experienced falls may be more inclined to internalize societal stereotypes that depict them as frail or dependent, potentially influencing their self-perception and emotional health. This internalization may exacerbate feelings of loneliness, as older adults might withdraw from social interactions due to fears of embarrassment or failure associated with their fall experiences (18).

A burgeoning body of research has attempted to establish an empirical link between falls and loneliness (19). Yet, three research gaps in the existing literature remain to be addressed. First, most of these studies have been conducted in Western contexts, such as the United States (20), Great Britain (21), and Germany (22, 23), while empirical evidence specifically focusing on older adults from non-Western and less developed societies such as China remains limited. Second, with only one exception (23) to the best of our knowledge, most previous studies have relied on cross-sectional designs (21, 22, 24, 25) which, in principle, fail to distinguish within-person changes from between-person differences, potentially leading to biased estimates. Third, little further attention has been paid to the potential heterogeneities, such as the disparities between the oldest-old and their younger counterparts, and mechanisms underlying the association between falls and loneliness, such as the mediating role of social adaptation.

Accordingly, this study aimed to extend prior research in three ways. First, to examine the general longitudinal association between falls and loneliness among Chinese older adults, we use data from a nationally representative longitudinal survey and the random-effects regression models. This focus on the Chinese context is particularly significant given China’s unique demographic transition, where population aging is occurring at an unprecedented pace and scale. Unlike Western societies that experienced gradual aging over many decades, China has undergone rapid demographic shifts due to its one-child policy and improved life expectancy, creating distinctive intergenerational dynamics and support structures (26). Moreover, traditional Confucian values emphasizing filial piety and family cohesion continue to shape eldercare practices in China, potentially buffering or exacerbating the psychological impacts of falls differently than in Western contexts. Following prior cross-sectional research (25), we expected that falls were related to higher levels of loneliness among older adults in China. Our findings would enrich our understanding of the psychological consequences of fall experiences among older adults in China and other countries that bears similar cultural traditions and are also experiencing rapid population aging.

Second, we examined whether the association between falls and loneliness would vary across age groups. Aging leads to various physiological changes (e.g., decreased strength, vision, and balance) that can increase the susceptibility to falls (27). In a cross-sectional study of older adults in China, the occurrence of falls for individuals in the 60–69 age group was 19.5%, increasing to 23.7% in the 70–79 age group, and further increasing to 29.2% for those in the ≥80 age group (27). Additionally, the levels of loneliness seem to increase with chronological age, especially within the older adults population. For example, one study based on a sample in Shanghai, China, found that the loneliness score was higher in the ≥80 age group than that in the 60–79 age group (28). With the rapid pace of population aging, the Chinese older population as a whole has expanded enormously and has also become increasingly diverse in terms of age, with distinctions emerging within the older population such as the young-old (aged 60–69), the old-old (aged 70–79), and the oldest old (aged 80 and over). In 2020, China had 264 million older adults aged 60 and above, among whom the young-old, the old-old, and the oldest old accounted for 55.83, 30.61, and 13.35%, respectively. Given that the oldest old often have higher rates of falls (27) and loneliness (5, 28), we expected that the impact of falls on loneliness would be more pronounced in the oldest old.

**Figure 1:**
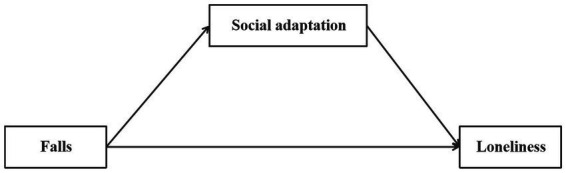
Conceptual diagram of the mediating pathway through social adaptation in the falls-loneliness relationship.

Third, we took a further step to investigate whether the association between falls and loneliness can be mediated by social adaptation. Social adaptation has often been defined as the “process of maintaining a positive mindset, accepting the realities of later life, and continuously making self-adjustments to adapt to the challenges of aging” (29, 30). On one hand, falls, as a unique negative life event during older adulthood, can be linked to compromised social adaptation. Theoretically, this is consistent with the stereotype threat model (16) because the internalization of stereotypes about older adults with fall experiences can lead to self-stigmatization and a shrinkage of social networks and social participation (31, 32), resulting in a decline in social adaptation. On the other hand, social adaptation has increasingly been recognized as a protective factor in shaping the psychological well-being of older adults (29, 33). For example, one recent study documented a positive link between social adaptation and mental health among Chinese older adults (29). Similarly, another study found a positive association between social adaptation and life satisfaction (33). Therefore, we hypothesized a mediation pathway whereby falls would negatively impact social adaptation, which in turn would lead to increased feelings of loneliness among older adults. Figure 1 illustrates the conceptual model depicting social adaptation as a potential mediator in the association between falls and loneliness. The diagram highlights two distinct pathways: (1) the direct pathway from falls to loneliness, represented by the lower horizontal arrow, and (2) the indirect pathway where falls affect social adaptation, which in turn influences loneliness, shown by the upper triangular path. This framework underscores how falls may exacerbate loneliness not only directly but also by undermining social adaptation capabilities, thereby elevating loneliness among older adults.

## 2 Data and methods

### 2.1 Data

Data were obtained from the China Longitudinal Aging Social Survey (CLASS), which is a nationally representative longitudinal survey of Chinese older adults aged 60 and above. In its baseline survey in 2014, CLASS used a multistage, area-based probability sampling design and collected 11,511 respondents from 462 rural villages/urban communities, 134 counties/districts, and 28 provinces in mainland China. Follow-up surveys were conducted every 2 years since the baseline. At each follow-up, a sample of newly eligible older adults was included to maintain the stability of the cross-sectional sample size. More details on the sampling design and data collection process can be found at http://class.ruc.edu.cn. This study was a secondary analysis based on publicly open dataset of CLASS. All studies were approved by the relevant Institutional Review Board. Ethical approval was not required for the analysis of the anonymized data. Informed consent was obtained from all participants involved in the study. To date, four waves of data from CLASS (2014, 2016, 2018, 2020) are available for public application.

**Figure 2:**
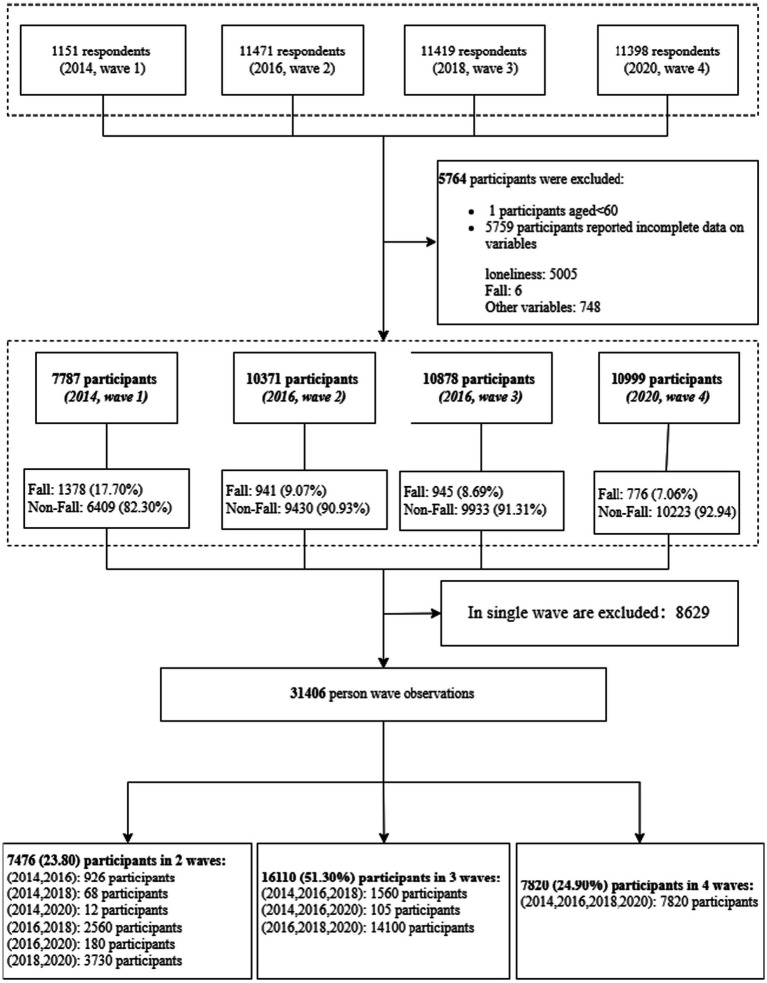
Flowchart of sample selection.

Based on our research objectives, we selected study samples that met the following two criteria: (1) had complete records of study variables, and (2) had at least two observation points. These procedures led to a final sample of 31,406 person-wave observations for 11,063 older adults. Figure 2 illustrates the step-by-step selection of eligible observations for analysis.

### 2.2 Measures

#### 2.2.1 Loneliness

Loneliness was assessed using a single item integrated in the Center for Epidemiological Studies Depression Scale (CES-D) (34), with respondents reporting how frequently they had felt lonely during the past week (0 = none of the time, 1 = some of the time, 2 = often) (26, 35, 36). Despite the existence of several standardized scales such as the UCLA Loneliness Scale (37) and the de Jong Gierveld Loneliness Scale (38), a single-item instrument remains the most commonly used in assessing loneliness (2), and bears moderate correlation with the standardized scales (39). Following the common practice of prior studies (26, 35), we treated loneliness as a continuous measure within a linear regression framework. Despite the ordinal nature of the original loneliness responses, treating them as continuous captures meaningful variations in loneliness intensity while simplifying the presentation of analytical results.

#### 2.2.2 Falls

Falls were evaluated using a single question: “Have you ever fallen in the past 12 months?” The response options included “No,” “Yes, only once,” and “Yes, twice or more.” Following prior practices (31, 40), we collapsed the latter two categories, obtaining a dichotomous variable distinguishing between older-age respondents with falls and those without falls. Such a dichotomization measure, allowing us to compare “fallers” to “non-fallers” in principle, has also been justified in previous research indicating no differences in health outcomes between experiencing single versus multiple falls (32).

#### 2.2.3 Social adaptation

Social adaptation, the focused mediator in the current study, was assessed using the Social Adaptation Scale developed by Chen (41). This scale had found wide application in empirical research across various fields (29, 42). The scale consists of eight items measuring different aspects of social adaptation: willingness to participate in community work, desire to contribute to society, learning enthusiasm, perceived social value, adaptation to social changes, acceptance of new opinions, response to social policies, and perception of age-friendly social changes. Respondents rated each item on a 5-point Likert scale ranging from “totally disagree” (=1) to “totally agree” (=5). The first four items were positively worded (e.g., “I am willing to engage in certain work in the village/community if given the opportunity”). The last four items were negatively worded (e.g., “The growing number of new social policies makes it harder for me to adjust”). Responses to negatively worded items were reverse-coded. All eight items were summed to obtain a total score. The total score ranged from 8 to 40, with higher scores reflecting greater social adaptation ability among older adults. In the current study, the scale showed higher internal reliability, with a Cronbach’s alpha of 0.71 at T1.

#### 2.2.4 Covariates

The analysis included demographic, family and socioeconomic characteristics, and health status, which have often been adjusted in prior research (31, 40). Demographic factors included gender, age and birth cohort groups. Family and socioeconomic characteristics included respondents’ educational attainment, marital status, living arrangement, residential region, Hukou and work status. Health status was assessed by chronic conditions, being coded 1 for having at least one type of chronic disease (e.g., hypertension, diabetes, etc.) and 0 for having no chronic disease. Finally, all regression models included a set of year dummies indicating the corresponding survey years to capture year variations. Detailed categories for each covariate can be found in Table 1.

**Table 1:** Descriptive statistics of sample characteristics (CLASS, 2014–2020).

		Age			*p*-value
	Total	60–69	70–79	≥80	
	*N* = 31,406	*N* = 16,028	*N* = 11,376	*N* = 4,002	
Loneliness (0–2)	0.54 (0.67)	0.49 (0.65)	0.57 (0.67)	0.64 (0.70)	<0.001
Falls %					<0.001
No	90.65	92.37	89.70	86.46	
Yes	9.35	7.63	10.30	13.54	
Gender %					<0.001
Male	51.45	52.48	50.78	49.25	
Female	48.55	47.52	49.22	50.75	
Cohort %					<0.001
Before 1935	5.92	0.02	0.23	45.73	
1935–1939	10.00	0.01	10.66	48.10	
1940–1944	16.07	0.58	41.40	6.10	
1945–1949	23.89	17.34	41.53	0.00	
1950 and after	44.12	82.04	6.18	0.07	
Educational attainment %					<0.001
Illiterate	22.68	16.98	25.64	37.06	
Primary	40.66	39.21	43.63	38.03	
Junior high	24.29	30.02	19.45	15.07	
High school and above	12.38	13.79	11.28	9.85	
Marital status %					<0.001
Married	72.82	82.37	68.91	45.68	
Widowed	25.66	15.54	30.12	53.57	
Divorced or never married	1.52	2.09	0.98	0.75	
Living arrangement %					<0.001
Living alone	11.31	8.14	13.30	18.34	
Living with only a partner	51.08	56.67	50.12	31.41	
Living with a partner and children	19.96	23.30	17.31	14.12	
Living with children	15.64	10.09	17.17	33.53	
Other living arrangements	2.01	1.79	2.10	2.60	
Hukou %					<0.001
Rural	56.61	57.51	57.30	51.10	
Urban	43.39	42.49	42.70	48.90	
Residence area %					<0.001
Downtown	36.37	36.40	35.1	39.86	
Suburb	9.82	10.09	9.57	9.42	
Combined urban–rural area	7.41	7.52	7.35	7.12	
Township	4.24	4.32	4.44	3.32	
Village	42.17	41.67	43.54	40.28	
Having paid employment %					<0.001
No	78.55	73.02	82.52	89.38	
Yes	21.45	26.98	17.48	10.62	
Chronic category conditions %					<0.001
No	30.81	36.49	26.13	21.39	
Yes	69.19	63.51	73.87	78.61	
Year %					<0.001
2014	9.96	11.84	8.30	7.15	
2016	28.79	32.32	25.58	23.79	
2018	33.33	32.92	32.51	37.36	
2020	27.91	22.92	33.61	31.71	

### 2.3 Statistical analysis

First, descriptive statistics were reported for the overall sample and the three age groups: the young-old, the old-old, and the oldest old. Means and standard deviations for continuous variables and percentages for categorical variables. To examine the longitudinal association between falls and loneliness, random-effects regression models were employed by including a person-specific error term to the conventional model, thereby accounting for the correlation across repeated observations within each respondent. We selected random-effects over fixed-effects models because this approach allowed us to estimate both within-person temporal changes and between-person differences simultaneously, while efficiently utilizing all available data and maintaining statistical power for time-invariant covariates in our longitudinal analysis. To investigate the potential moderating effect of age on the relationship between falls and loneliness, we further constructed interaction terms between falls and the age group dummies which were then included in our regression model. Finally, to explore the potential role of social adaptation in mediating the association between falls and loneliness, we employed the Baron and Kenny method (43) and Bootstrapping procedures to obtain the significance and magnitude of the indirect effect of falls on loneliness via the pathway of social adaptation. The Baron-Kenny approach involves a series of regression models to establish: (1) a significant association between the independent variable (falls) and the dependent variable (loneliness), (2) a significant relationship between the independent variable and the proposed mediator (social adaptation), and (3) whether the mediator significantly predicts the dependent variable when controlling for the independent variable. Mediation is supported if the effect of falls on loneliness is attenuated upon the inclusion of social adaptation in the model. Additionally, we used nonparametric Bootstrapping procedures (with 5,000 resamples) to directly estimate the confidence intervals for the indirect effect. This method does not assume normality of the indirect effect distribution and provides a more robust assessment of mediation by generating empirical sampling distributions and bias-corrected confidence intervals. A significant mediation effect is indicated if the confidence interval for the indirect effect does not include zero. Throughout all regression models, coefficients along with 95% confidence intervals were reported. Statistical significance was defined as *p* < 0.05 for all analyses. Finally, all analyses were performed using Stata for Windows, version 12.0 (StataCorp, College Station, TX, United States).

## 3 Results

### 3.1 Descriptive results

Table 1 shows the characteristics of respondents in the pooled sample and subsamples by age group. In the total sample, the mean loneliness of older adults is 0.54 (SD = 0.67), and 9.35% of older adults had reported at least one fall in the past 12 months. In terms of age subsample characteristics, there were significant differences in falls. Specifically, compared with the young-old (aged 60–69), the old-old (aged 70–79) reported a higher fall experience (7.63% vs. 10.30%), and the oldest old (aged 80+) reported the highest fall experience (13.54%). The prevalence of loneliness also increases significantly with age, consistent with prior research (21, 25, 28).

### 3.2 Regression results

Table 2 presents the results of random effects regression models of the association between falls and loneliness among older adults in China. With either unadjusted (Column 1) or adjusted (Column 2) covariates, the coefficients for falls are consistent. After adjusting variables, including sex, age group, birth cohort, education, marital status, living arrangement, hukou, residence, employment status, chronic disease, and survey year dummy, falls were significantly associated with loneliness (*β* = 0.061, 95% CI = [0.035, 0.086], *p* < 0.001).

**Table 2:** Estimated effect for loneliness by fall, CLASS 2014–2020.

	Model 1	Model 2	Model 3
	Loneliness score	Loneliness	Loneliness
Falls	0.085^***^	0.061^***^	0.018
	[0.060, 0.110]	[0.035, 0.086]	[−0.021, 0.057]
Age (ref. 70–79)			
60–69		−0.009	−0.014
		[−0.035, 0.017]	[−0.040, 0.013]
80+		−0.003	−0.018
		[−0.042, 0.035]	[−0.058, 0.022]
Age # falls			
Age (60–69) # falls			0.053^*^
			[0.000, 0.107]
Age (80+) # falls			0.112^**^
			[0.043, 0.181]
Gender (female = 1)		−0.007	−0.007
		[−0.026, 0.011]	[−0.025, 0.011]
Birth cohort (ref. before 1935)			
1935–1939		0.014	0.015
		[−0.034, 0.060]	[−0.032, 0.062]
1940–1944		−0.015	−0.014
		[−0.071, 0.041]	[−0.069, 0.041]
1945–1949		−0.027	−0.028
		[−0.085, 0.030]	[−0.085, 0.029]
1950 and after		−0.017	−0.017
		[−0.079, 0.045]	[−0.080, 0.045]
Other covariates	Unadjusted	Adjusted	Adjusted
Observations	31,406	31,406	31,406

95% confidence intervals in brackets. ^*^p < 0.05, ^**^p < 0.01, ^***^p < 0.001.

To further examine how age moderates the association between falls and loneliness, we included interactions between falls and two age-group dummies in our model. As shown in Column 3 of Table 2, the interaction terms between falls and the young-old and between falls and the oldest-old both showed statistical significance in predicting loneliness compared to the old-old, indicating that the association between falls and loneliness is stronger among the young-old and the oldest-old compared to the old-old.

### 3.3 Mediating effect result

Table 3 shows the mediating role of social adaptation in the falls-loneliness relationship. Model 1 shows that falls significantly positively predicted loneliness among older adults (*β* = 0.061, 95% CI = [0.035, 0.086], *p* < 0.001). Model 2 shows that falls significantly negatively predicted social adaptation (*β* = −1.017, 95% CI = [−1.252, −0.782], p < 0.001). In addition, Model 3 shows that social adaptation has a significant negative effect on loneliness (*β* = −0.007, 95% CI = [−0.008, −0.005], *p* < 0.001), and fall has a significant positive impact on loneliness (*β* = 0.054, 95% CI = [0.029, 0.079], *p* < 0.001). Notably, the coefficient of falls in Model 3 was reduced compared to Model that reported in Model 1. Furthermore, the results based on bootstrap mediating analysis reveal that the indirect effect of fall on loneliness through social adaptation was significant (*β* = 0.006, 95% CI = [0.004, 0.009], *p* < 0.001). Specifically, the indirect path (Fall→Social adaptation→Loneliness) accounted for 10% of the total effect. Therefore, the mediating effect of social adaptation was further confirmed.

**Table 3:** Mediation effects test results (mediating variable: social adaptation).

	Model 1	Model 2	Model 3
	Loneliness	Social adaptation	Loneliness score
Falls	0.061^***^	−1.017^***^	0.054^***^
	[0.035, 0.086]	[−1.252, −0.782]	[0.029, 0.079]
Social adaptation			−0.007^***^
			[−0.008, −0.005]
Control variables	Controlled	Controlled	Controlled
Constant	0.482^***^	22.488^***^	0.629^***^
	[0.414, 0.549]	[21.858, 23.119]	[0.557, 0.702]
Bootstrap	β	95% CI	% of total
Indirect effect	0.006^***^	[0.004 0.009]	10%
Direct effect	0.054^***^	[0.029 0.079]	
Total effect	0.060^***^	[0.035 0.086]	
Observations	31,406	31,406	31,406

95% confidence intervals in brackets. ^*^p < 0.05, ^**^p < 0.01, ^***^p < 0.001.

## 4 Discussion

Falls and loneliness are both common among older adults, with the former being an unexpected negative life event and the latter being a typical mental health problem in older adulthood. Despite the considerable research attention paid to the psychological consequences of falls and the various determinants of loneliness, there is still a lack of research specifically investigating whether and how falls may be longitudinally related to loneliness among older adults, especially in China. Based on data from a nationally representative longitudinal survey of Chinese older adults, this study used random-effects regression models to examine the longitudinal association between falls and loneliness, the age disparities in the association, and the role of social adaptation in mediating the association.

First, extending much prior work documenting an cross-sectional association between falls and loneliness in both Western and Chinese contexts (22, 25), we found that falls were longitudinally linked to increased levels of loneliness among older adults in China. This finding aligns with a cross-sectional study in Germany which, based on cross-sectional data from a population-based sample, showed that experiencing a fall in the past year was associated increased loneliness (22). In contrast, a more recent study in Germany with longitudinal data, which was based on community-dwelling individuals aged 40 and over, failed to detect a significant association between falls and loneliness. Since this study included both mid-aged older adults, the longitudinal association between falls and loneliness among German older adults remains unknown. In the context of China, several recent studies have established that falls and fall-related injuries were related to greater psychological symptoms such as depression and anxiety (25, 27, 35, 44). However, few previous attempts have been made to specifically examine the association between falls and loneliness among Chinese older adults. Notably, a recent study based on cross-sectional data in China observed that a fall experience in the past 3 months was associated with increased loneliness among older adults aged 60 and above (25), which is consistent with our finding. However, the longitudinal association between falls and loneliness and the heterogeneity of the association were beyond the scope of this prior study.

Second, we examined whether the association between falls and loneliness might vary across different age segments of Chinese older adults (i.e., the young-old, the old-old, and the oldest old). We found that the association did vary across age groups, not monotonically, but in a more interesting way. Specifically, compared to its association with loneliness in the old-old, the associations of falls with loneliness were more pronounced in the young-old and the oldest old. A plausible explanation is that falls are comparatively less common among the young-old, and thus the occurrence of falls may be unexpected and even unaccepted as a “normative” event (45), leading to high levels of psychological symptoms including loneliness. From the perspective of the cognitive dissonance theory (46), individuals experience mental discomfort when holding inconsistent cognitions. For the young-old, who may perceive themselves as physically capable and far from frailty, a fall introduces a profound contradiction between their self-perception and the reality of the event. This discrepancy between their expected robust behavior and the unexpected fall generates significant unease, motivating a reduction of this dissonance which can manifest as increased psychological distress. On the other hand, falls among the oldest old may be comparatively more severe in terms of associated physical injuries and functional limitations (13), thereby leading to greater loneliness. To the best of our knowledge, no empirical research has explicitly explored the age disparities in the association between falls and loneliness.

Third, we examined the potential mediating role of social adaptation in channeling the effect of falls on loneliness. Our mediation analysis revealed that social adaption can account for a partial proportion (about 10%) of the association between falls and loneliness. While this mediating effect is small to moderate in magnitude (43), it represents a statistically significant pathway through which falls influence loneliness, highlighting social adaptation as one of multiple mechanisms that contribute to this relationship. As an essential part of achieving active aging (33), social adaptation has increasingly been found to be beneficial for the psychological well-being of older adults (29, 33). Falls have also been found to lead to decreased social participation and adaptation (31, 44). Our findings contributed to the prior literature by demonstrating that falls can lead to lower levels of social adaptation which in turn can lead to higher levels of loneliness.

Our findings offer several important policy implications. First, falls prevention programs should be integrated into broader mental health initiatives for older adults, recognizing falls not just as physical health events but as potential triggers for loneliness and social isolation. Public health campaigns could raise awareness about this connection, while healthcare providers should incorporate loneliness screening into post-fall assessments and follow-up care. Second, our age-stratified analysis reveals that both the young-old and the oldest-old represent particularly vulnerable subgroups among Chinese older adults, suggesting the need for age-tailored interventions rather than a one-size-fits-all approach. For the young-old, programs might focus on maintaining social roles and identity during the transition to retirement, while for the oldest-old, more intensive support systems may be needed to address multiple health challenges and declining social networks. Third, the mediating role of social adaptation highlights an actionable pathway for intervention. Community-based programs could facilitate adaptive strategies for older adults with fall histories, such as accessible transportation services, home modification initiatives, and technology-assisted social engagement opportunities. Additionally, training programs for family caregivers and healthcare professionals should emphasize techniques to support older adults in maintaining social connections after experiencing falls.

Several limitations must also be acknowledged. First, the use of self-reported data to document falls in older adults is inherently limited, primarily because of recall bias. Participants may forget incidents or inaccurately report falls that occurred outside of the specified time frame. This recall bias could be particularly pronounced among the oldest-old participants who may experience cognitive decline, potentially leading to underestimation of fall incidence in this vulnerable group. Our binary assessment of falls also cannot capture recurrent falls as well as their association with loneliness. The use of alternative research methods in future studies, such as hospital administrative records (47) or mobile device applications, may provide more accurate tracking of falls by allowing timely data collection. Second, we relied on a single item to measure loneliness, which may not capture the multiple dimensions of loneliness. Future research can integrate more comprehensive assessment of loneliness, such as such as the UCLA Loneliness Scale, into data collection and analysis. Third, the association between falls and loneliness demonstrated in this study should be interpreted with caution due to the potential for reverse causation. Despite the theoretical and empirical support for the impact of falls on loneliness, there is also evidence that older adults who feel lonely are more likely to experience falls (47–49). Although our longitudinal data analysis can mitigate data dependency issues, the possibility of reverse causation cannot be completely excluded. To better establish causality, future research can use intra-individual fixed-effect methods or instrumental variable approach with appropriate data. Fourth, the exclusive exploration of social adaptation as a mediator in the current study leaves open the question of whether social adaptation can effectively moderate the association between falls and loneliness among older adults. Such an investigation, although beyond the scope of the current study, certainly merits attention in future research. Finally, the findings in the current study were based on a Chinese sample and thereby may not generalize to other cultural or ethnic groups.

In conclusion, this study demonstrates that falls significantly contribute to loneliness among older Chinese adults, particularly in the young-old and oldest-old age groups, and that social adaptation partially mediates this relationship. These findings enrich our understanding of the psychological consequences of falls among older adults in China and other rapidly aging societies.

## Data Availability

Publicly available datasets were analyzed in this study. This data can be found at: The data that support the findings of this study are available from CLASS project site, subject to registration and application process. Further details can be found at: http://class.ruc.edu.cn. We agreed to share the data from this study if we obtain permission from the CLASS project.
